# Induction of Apoptosis in Cancer Cells of pre-B ALL Patients after Exposure to Platelets, Platelet-Derived Microparticles and Soluble CD40 Ligand 

**DOI:** 10.22074/cellj.2018.5032

**Published:** 2018-01-01

**Authors:** Morteza Yaftian, Fatemeh Yari, Mehran Ghasemzadeh, Vahid Fallah Azad, Mansoureh Haghighi

**Affiliations:** 1 Blood Transfusion Research Center, High Institute for Research and Education in Transfusion Medicine, Tehran, Iran; 2 Mahak Pediatric Cancer Research and Hospital Center, Tehran, Iran

**Keywords:** Acute Lymphoblastic Leukemia, Apoptosis, CD40 Ligand, Microparticles, Platelet

## Abstract

**Objective:**

The *in vitro* treatment of tumor cells with platelet (Plt) causes inhibition of tumor cell growth, although mechanism of this effect is not clear yet. Induction of apoptosis has been proposed as a mechanism of Plt effects on tumor cells. The purpose of this study was to clarify the role of Plts and Plt-derived components in the induction of apoptosis in the blood mononuclear cells of patients with leukemia.

**Materials and Methods:**

In this experimental study, peripheral blood mononuclear cells (PBMCs) were isolated from whole blood of five patients with childhood B-precursor acute lymphoblastic leukemia (pre-B ALL) and encountered with Plts, Plt-derived microparticles (Plt-MPs) as well as purified soluble CD40L (sCD40L). After 48 hours of co-culture, the anti-cancer activity of the aforementioned factors was surveyed using examination of apoptosis markers of the cells including active caspase-3 and CD95 using ELISA and flow cytometer techniques, respectively. Additionally, staining of the cells with 7-Aminoactinomycin D (7-AAD) was evaluated by flow cytometer technique. Trypan blue exclusion test and WST-1 method were also used to compare the death/survival status of the cells.

**Results:**

Levels of CD95 and caspase-3 were significantly increased in the all treated groups (P<0.05). On the other hand, trypan blue, 7-AAD and WST-1 methods showed significantly lower number of the live cells in the treated groups (P<0.05).

**Conclusion:**

This study can show the ability of Plts, Plt-MPs and sCD40L for the induction of apoptosis in PBMCs of pre-B-ALL patients. Further studies are necessary to elucidate the different effects of platelets on cancer cells *in vitro* and *in vivo*.

## Introduction

Platelets (Plts) are created by cytoplasmic destruction of megakaryocytes (1). Each megakaryocyte is likely to produce 2000-5000 nascent Plts (2). In addition to their fundamental role in homeostasis, Plts contain both angiogenesis and angiostatic compounds. These proteins are arranged in a-granules and can be secreted differentially by selective stimulation of thrombin receptors, PAR-1 and PAR-4 (3, 4). Once Plts are stimulated or exposed to high shear stress, they release particles expressing membrane receptors and cytoplasmic components that are named Plt-derived microparticles (PLT-MPs) (5). 

PLT-MPs express surface proteins and chemokine receptors which could be transferred to the surrounding cell membranes (6, 7). Plts are composed of many biologically active proteins within their cytoplasmic granules including a-granules, dense granules and peroxisomes (8). They contain abundant CD40L in their alpha-granules and upon activation they express CD40L on their surface or release soluble CD40L proteins into the medium. Activated Plts also express other molecules on their surface such as P-selectin, FC receptor for immunoglobulin IgG and Fas L (9, 10). Abundant quantities of Fas L (CD95 L) are existed in the a-granules of human Plts. Upon activation, Plts express Fas L on their surface as well as releasing it into the medium (11). 

It has been determined that some cancer cells are able to trigger Plts (12). Human cancer cells can directly attach to Plts and activate it through α3-integrins of cancer cells and surface molecules, like glycoprotein IIb/IIIa on Plts, or through releasing mediators such as ADP, thromboxane A2 or tumor-associated proteinases (13). Besides, it is known that Plts directly exert cytotoxic effects against certain human tumor cells (14, 15) or inhibit tumor cell growth via the cell cycle arrest (16). Pre-B ALL is an aggressive cancer of immature B cells with the virtue of cytoplasmic immunoglobulin positivity. In this study, we evaluated the ability of human Plts, Plt-MPs and soluble CD40L (sCD40L) for the apoptosis induction in peripheral blood mononuclear cells (PBMCs) of pre-B ALL patients. The results of this study can be useful for showing the apoptosis induction potential of human Plts, Plt-MPs and sCD40L on tumor cells *in vitro* using cells derived from patients with Pre-B ALL.

## Materials and Methods

In this experimental study, five single donor Plt concentrate bags (JMS Singapore Pte Ltd. containing CPDA-1 solution, Singapore) were obtained from Iranian Blood Transfusion Organization (IBTO, Iran) after concentrating Plts and performing viral safety tests. Informed consent was acquired from the blood candidates by IBTO. Plts were aliquoted into 15 ml conical centrifuge tubes and centrifuged at 300 g for 5 minutes, to remove red blood cells (RBCs) and white blood cells (WBCs). The supernatant was centrifuged again at 1200 g for 10 minutes to sediment Plts. Plts were washed three times with phosphate-buffered saline (PBS), then resuspended in RPMI 1640 medium (Sigma-Aldrich, USA). They were subsequently enumerated by using an automated hematology analyzer (Sysmex K-1000, Japan). Additionally, Plts were activated in presence of the thrombin (3 U/ml) at room temperature.

### Isolation and characterization of microparticles 

Plt-MPs were prepared from Plt concentrate at the third day of storage. Plt concentrate was centrifuged at 1200 g for 15 minutes to sediment RBCs, WBCs and Plts. Then the supernatant was centrifuged at 16000 g for 15 minutes to precipitate Plt-MPs. The pellet was washed three times with PBS to eliminate the plasma proteins. Eventually, the concentration of Plt-MPs was measured by Bradford method. The size range of Plt-MPs was determined by flow cytometer technique with the CyFlow®Space system (Partec, Germany) using 1 µm fluorescent microbeads; FluoSpheres® microspheres (molecular probes, USA).

### Purification and characterization of CD40-ligand 

An affinity chromatography column was prepared with covalent attachment of anti-CD40L with Cyanogen bromide-activated agarose matrices (Sigma, USA). After elimination of RBCs, WBCs, Plts and Plt-MPs from Plt concentrate, plasma was passed through the anti-CD40L monoclonal antibody affinity column, using high speed centrifugation (16000 g). Washing step was carried out using PBS to remove unattached proteins. CD40L was eluted by the elution buffer (0.1 M glycine, pH=2.8). A Spin-X® UF 20 (5K MWCO) concentrator tube (Corning, UK) was used to concentrate the purified protein. Concentration of the purified protein was determined with Bradford method and specificity of the protein was confirmed by western blotting, as described below. Thirty microliter of the purified protein was subjected to 12% sodium dodecyl sulfate-polyacrylamide gel electrophoresis (SDS-PAGE). Proteins were separated on SDS-PAGE gel and electrophoretically transferred to Polyvinylidene difluoride (PVDF) membrane. The blot was incubated with 1:150 dilution of primary antibody (anti-CD40L; Abcam, UK). After the washing step, the blot was incubated with 1:1000 dilution of HRP- labeled secondary antibody (anti-mouse IgG, Abcam, UK). Antibody binding was detected using enhanced chemiluminescence (ECL) and quantified in a Biorad scanner (Biorad chemidoc XRS system, USA) using Image Lab Tm.

### Selection of patients 

Five Pre-B ALL patients (3-7 years old) were diagnosed in Tehran Mahak Hospital (Iran) based on the immunophenotyping analysis using flow cytometer technique and other clinical/ experimental evidences. The patients had no previous treatment. Any medication and drug receiving were the exclusion criteria of this study. The samples were attained after obtaining informed consent under the Ethical principles and the protocols approved by Mahak Pediatric Cancer Research and Hospital Center, Tehran. 

### The co-culture process

PBMCs were obtained from the whole blood of patients by density gradient centrifugation using Ficoll-Paque (Innotrain, Germany). Cells were washed three times with PBS, suspended in RPMI 1640 and counted with an automated hematology analyzer. Unstimulated and thrombin-activated Plts (5×10^7^ cells), 200 µg/ml Plt-MPs and 50 µg/ml CD40L were separately introduced to the culture medium included PBMCs (1×10^5^ cells) of pre-B ALL patients in the wells of a cell culture plate. The co- culture medium consisted of RPMI 1640 supplementing with 10% FBS (Invitrogen, USA), 1% penicillin and streptomycin (containing 10,000 IU/ml penicillin and 10,000 ug/ml of streptomycin, Gibco, USA) and 200 mM L-glutamine (Sigma, USA). The culture plates were incubated at 37°C for 48 hours in a humidified incubator with 5% CO_2_ atmosphere. It is worthy of note that all of the treatments and co-cultures were performed in duplicate and appropriate controls were used for each run. Control wells were consisted of PBMCs without any treatment.

### 7-amino actinomycin D viability staining 

7-amino actinomycin D (7-AAD) is a fluorescent intercalator that undergoes a spectral change upon interaction with DNA. 7-AAD/DNA complexes can be excited by the 488 nm laser and has an emission maximum of 647 nm. After the co-culture time, cells in each well were harvested and centrifuged at 400 g for 5 minutes to remove Plts. Deposited cells were washed once with assay buffer (Cayman 7-AAD assay kit, USA), resuspeneded in the staining solution of the kit and kept at room temperature for 15 minutes at dark. Cells were centrifuged at 400 g for 5 minutes and suspended in assay buffer for analysis with flow cytometer technique.

### CD95 expression levels

The expression level of CD95 was studied on PBMCs of B-ALL patients after the co-culture time by flow A cytometer technique. In a one-step method, 2 µl of FITC- conjugated mouse anti-CD95 antibody (eBioscience, USA) was added to the tubes, each of which included 1×10^5^ cells in 100 µl. The tubes were left for 40 minutes at 4°C before analysis by flow cytometer.

### Active caspase-3 concentration

The experiment was based on the measurement of P17 subunit of active caspase-3 inside the human cells using ELISA method (Abcam, UK). Forty-eight hours after the co-culture time, PBMCs of patients were lysed in the extraction buffer included in the kit after addition of the protease inhibitor [1 mM phenylmethyl sulfonyl fluoride (PMSF)]. The extracted protein solutions were B stored at -80°C. ELISA method was done according to the manufacturer’s instructions.

### WST-1 cell proliferation and viability assay 

WST-1 is a method to measure cell proliferation and based on the cleavage of the tetrazolium salt (WST-1) to formazan by cellular mitochondrial dehydrogenase enzymes. Expansion in the number of viable cells results in an increase in the activity of the mitochondrial dehydrogenases, which in turn leads to increase in the amount of the formed formazan dye. After co-culture time, cells were centrifuged at 400 g to remove Plts. Next, 100 µl of resuspended cells were seeded in a 96 well flat bottom plate. Subsequently, 10 µl of WST-1 reagent (Cayman cell proliferation assay kit, USA) was added to each well. The plate was incubated for 4 hours at 37°C in a humidified incubator and 5% CO_2_ atmosphere. The absorbance of wells was measured with a microplate absorbance reader (Asys Expert 96, UK) at the wavelength of 450 nm. 

### Trypan blue exclusion test of cell viability

Trypan blue is a vital stain, providing a possibility for detection of live and dead cells, microscopically. After the end of co-culture time, numbers of viable and dead cells were enumerated for the cells of each well of culture plate by Trypan blue. Finally, the percent of dead cells was calculated.

### Statistical methods

The non-parametric; Wilcoxon method with SPSS 16.0 software was used to analyze the results of this research. The P<0.05 was considered statistically significant. 

## Results

### Characterization of microparticles

**Fig.1:**
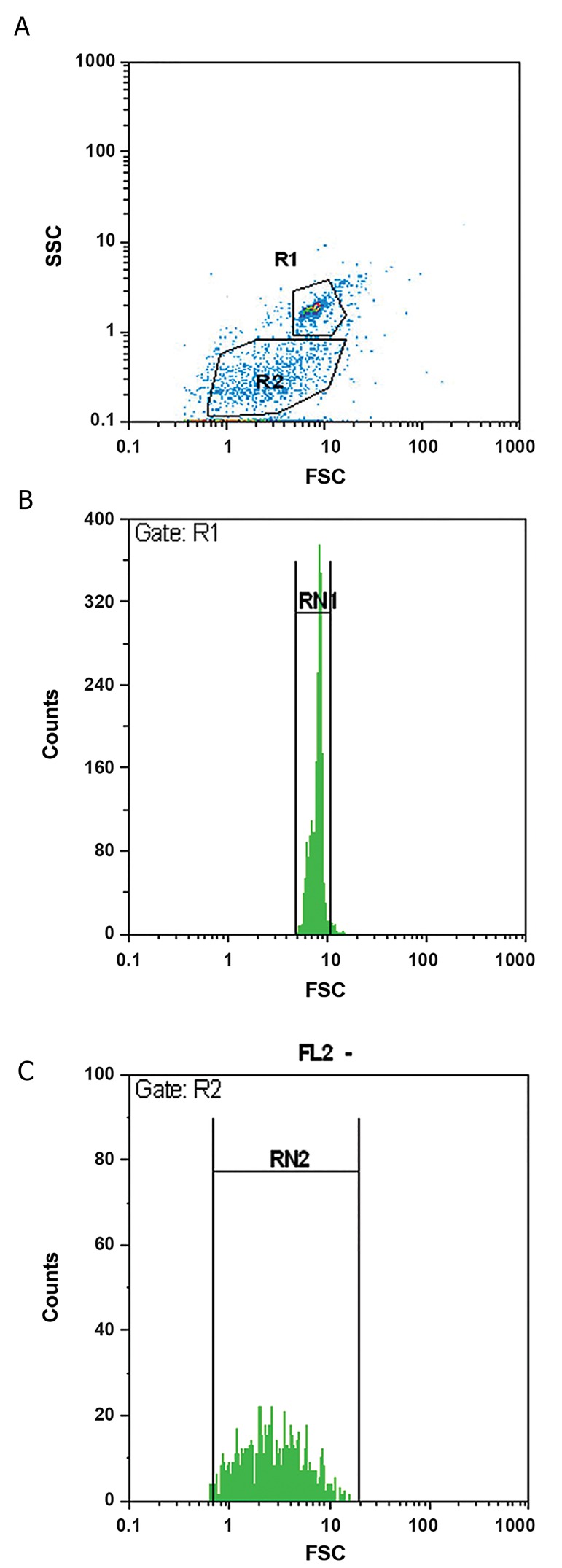
Flow cytometer plot and size distribution of platelet microparticles. Comparison of the size of fluorescent microspheres (1 µm microbeads) and unknown sized microparticles. Images represent, A. Gating of the microbeads (R1) and microparticles (R2), B. Diagram for the size range of microbeads, and C. Diagram for the size range of microparticles. The figure shows a smaller size and more heterogeneity in the size for platelet microparticles compared to 1 µm microbeads.

The isolated MPs had less than 1 µm diameter. Additionally, a heterogeneous population differing in the size was demonstrated for the isolated MPs using microbeads (Fig .1). The platelet origin of MPs was confirmed by flow cytometer using FITC-conjugated antiCD41 antibody (data not shown).

### Characterization of the purified soluble CD40L 

**Fig.2:**
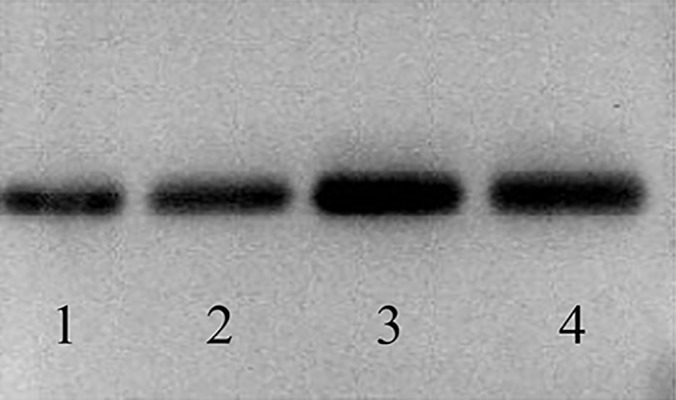
Soluble CD40L was purified from platelet concentrate by affinity chromatography using anti-human CD40L column. The specificity of CD40L was confirmed by western blotting using specific monoclonal antibody to CD40L. The results are represented for the purified samples. 1, 2, 3 and 4 referred to different lots of purified soluble CD40L.

The specificity of the purified protein was shown by western blotting. The protein reacted well with antiCD40L antibody (Fig .2). The concentration of the protein (µg/ml) was determined by Bradford method after protein condensing.

### 7-amino actinomycin D viability staining results

**Fig.3:**
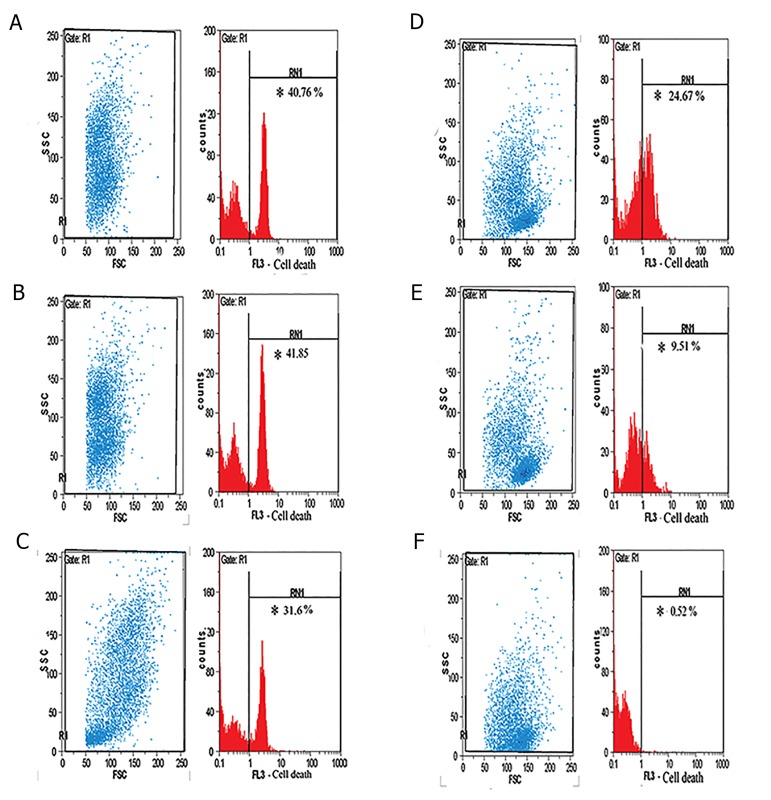
Flow cytometer plot for 7-AAD staining. The gating and percentage of 7-AAD staining for peripheral blood mononuclear cells (PBMCs) of pre-B acute lymphoblastic leukemia patients after co-culture with: A. Plt, B. Thrombin-activated platelets (aPlt), C. Platelet-derived MPs, D. sCD40L, E. Control cells (PBMCs alone), and F. unstained PBMCs. *; Percentage of the dead cells after the co-culture time and MPs; Microparticles.

Percentage of 7-AAD-stained cells was measured by flow cytometer technique (Fig .3) and showed significantly greater percent in the treatment groups than the control group (P=0.04).

### CD95 expression level

**Fig.4:**
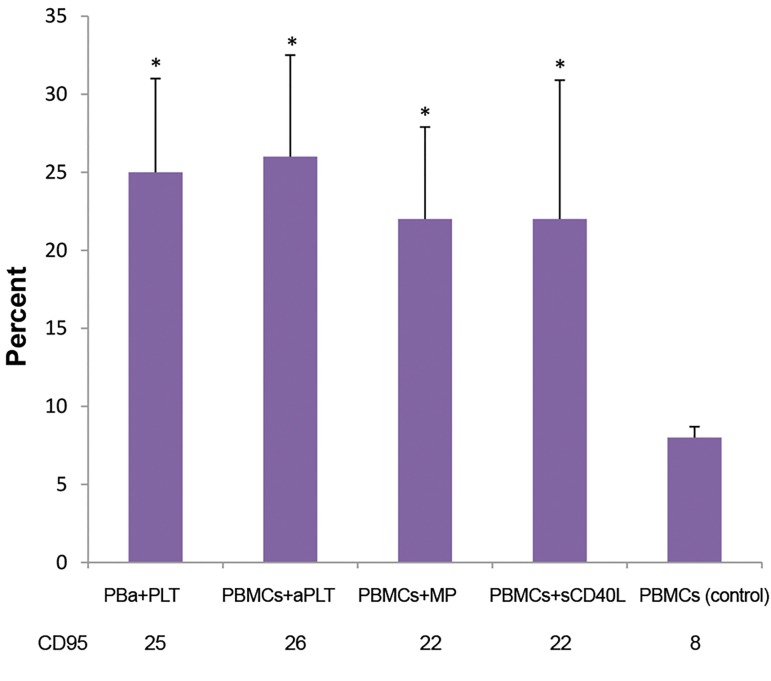
PBMCs of pre-B acute lymphoblastic leukemia patients were treated with each of the following factors: Plt, aPlt, platelet-derived MPs and sCD40L. Higher expression of CD95 was observed in all of the treated groups, compared to the control group. Data are presented as the mean ± SD of five independent experiments (*; P<0.05). PBMCs; Peripheral blood mononuclear cells, aPlt; Thrombinactivated platelets, and MPs; Microparticles.

CD95 (FAS) expression level was analyzed on PBMCs of pre-B-ALL patients after 48 hours co-culture, using the flow cytometer technique. A significantly higher expression of CD95 was detected in all of the treated groups, in comparison with the untreated control group (P=0.04, Fig .4).

### Active caspase-3 concentration

**Fig.5:**
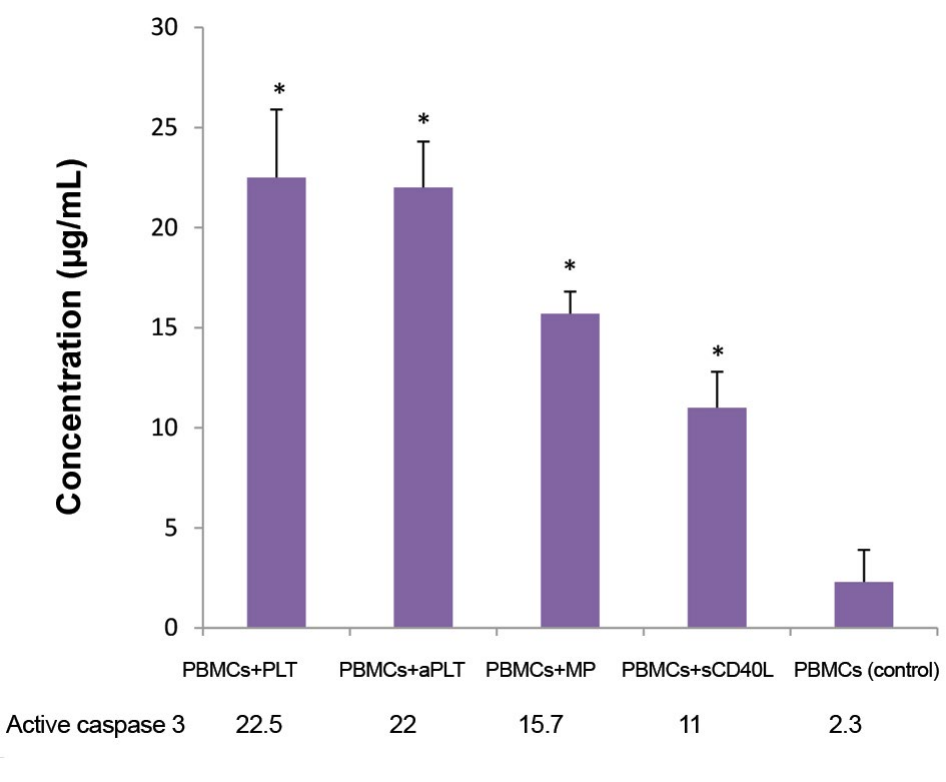
Active caspase-3 levels in PBMCs of pre-B acute lymphoblastic leukemia patients after the co-culture time. Higher concentrations of caspase-3 are shown due to the vicinity of PBMCs with each of the following factors: Plt, aPlt, platelet-derived MPs and sCD40L. Higher expression of active caspase-3 was observed in all the treated groups, compared to the control group. Data are presented as the mean ± SD of five independent experiments (*; P<0.05). PBMCs; Peripheral blood mononuclear cells, aPlt; Thrombinactivated platelets, and MPs; Microparticles.

In all of the treated groups, concentration of active caspase-3 was significantly increased in the extracts of leukemia cells after the co-culture time compared to the control group (P=0.043, Fig .5).

### Trypan blue and WST-1 results

**Fig.6:**
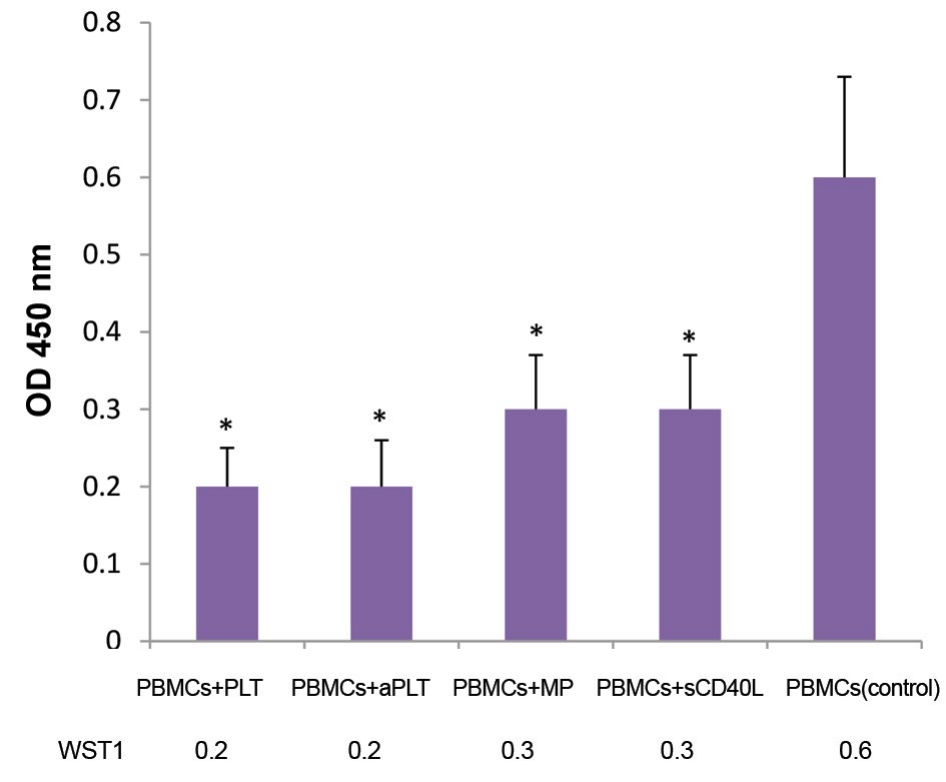
WST-1 results. Enzymatic cleavage of WST-1 to formazan by cellular mitochondrial dehydrogenases was measured by reading absorbance at the wavelength of 450 nm. Exposure to Plts, platelet-derived MPs or sCD40L caused lower viability and metabolic activity of the PBMCs of pre-B acute lymphoblastic leukemia patients. Lower survival was determined in all of the treated groups, compared to the control group. Data are presented as the mean ± SD of five independent experiments (*; P<0.05). PBMCs; Peripheral blood mononuclear cells, aPlt; Thrombin activated platelets, and MPs; Microparticles.

Plasma membrane of non-viable cells was permeable to Trypan blue whereas live cells remained unstained. The number of viable and dead cells was counted microscopically after the co-culture time and the percent of dead cells was calculated. Trypan blue showed a significantly increased number of dead cells after the treatments, in comparison with the untreated control cells. The highest decrease in the viability of patient cells were observed upon co-culture of PBMCs with Plts and the lowest decrease in the viability was observed for sCD40L-treated PBMCs (P=0.042). On the other hand, WST-1 showed lower viability and metabolic activity in PBMCs of patients after exposure to Plts, in comparison with PMP and sCD40L. The differences between each treated case and control was significant (P=0.04, Fig .6). 

## Discussion

Despite the role of Plts in tumor spread in the body (14, 17), cancer killing effects of Plts have been demonstrated using cell lines (15, 16). This experiment was done to evaluate the effects of Plts and Plt components on the cells of leukemia patients following *in vitro* treatment. PBMCs were obtained from the pre-B ALL patients and exposed to Plts, Plt-MPs and sCD40L. The viability and apoptosis of the cells were determined after the co-culture time. The results indicated that these factors could cause both growth inhibition and apoptosis of leukemic cells after 48 hours of co-culture.

Our investigation was consistent with some studies related to Plt impact on cell lines such as Ible (15), and Bykovskaya et al. (18), but with two main differences. Firstly, we used cells from patients with pre-B ALL instead of cell lines and secondly, in addition to Plts, we comparatively studied the effects of Plt-MPs and purified sCD40L on cancer cells.

The findings of this study disagreed with that of Wang and Zhang (16) reported, who stated that the inhibition of tumor cell growth by Plts was mainly due to the cell cycle arrest, but neither cytotoxicity nor apoptosis mechanisms. At present, we know that there is a relation between the cell cycle and apoptosis. This link arises from the collected evidence that manipulation of the cell cycle may either inhibit or induce an apoptosis (19). So it seems that Wang’s experiment do not rule out apoptosis in tumor cells due to the interaction with Plts. 

We also showed that both thrombin-activated Plts and unstimulated Plts have nearly similar influences on pre-B ALL cells. Correlated with us, Sagawa and co-workers showed similar results on tumor cells (20).

Active Plts release sCD40L over a period of minutes to several hours (21-23). In the present study, we used purified sCD40L in co-culture with leukemia cells. After 48 hours of co-culture, the results for sCD40L showed similar effects to Plts but in a lesser extent. Our results about the effects of sCD40L on cancer cells of pre-B ALL patients were concomitant with the studies of Kedar and co-workers who showed CD40 ligation may deliver signals that induce apoptosis and growth arrest in B-cell malignancies (24).

The present study also shows higher expression level of CD95 (FAS) on the studied cancer cells after treatment with Plts or Plt-derived components. Consistent with these findings, Afford et al. (25), Alexandroff et al. (26) and Qu et al. (27) found that CD40-CD40L ligation upregulates Fas expression on tumor cells. So, this effect may be related to soluble or cell surface CD40L and the enhanced FAS expression should predispose cells to apoptosis (28). In line with our findings Pellat-Deceunynck et al. (29) and Funakoshi et al. (30) showed the role of CD40 ligation on tumor cells of multiple myeloma and primary high-grade B-cell lymphoma, respectively.

Application of Plt-MPs was a main feature of our study and provided new data for comparing the anti-cancer features of Plt-MPs, sCD40L and platelets *in vitro*. Furthermore, the set of experiments that we performed and discussed here was directly related to apoptosis. 

## Conclusion

Plts and Plt-derived components -MPs and sCD40L (all from normal blood donors)have apoptotic effects on childhood pre-B ALL cells *in vitro*. Further studies are required to survey different effects of Plts or Pltderived components in tumor microenvironment *in vivo*. Differences may arise from different features of Plts in cancer patients.
